# Nepenthe

**Published:** 1864-06

**Authors:** 


### NEPENTHE.

"It is equal to any thing Mrs. Stowe has written," said a lady of culture and eminent critical talents, of this new volume of the writer of *Olie*. For the truthfulness, beauty, and purity of its sentiments, few works of fiction have been produced at home or abroad. It will be eagerly read by the physician, the lawyer, and the divine; for the talented writer who has worked out this beautiful narrative seems to be at home in all these vocations; is equally expert in setting a broken bone, in unraveling a knotty point in law, and making clear as the light of day the principles and practice which will secure pulpit success. We commend the book to the learned professions.

